# Hyperspectral Retinal Imaging as a Non‐Invasive Technique to Determine Brain Amyloid Status

**DOI:** 10.1002/alz70856_105392

**Published:** 2026-01-10

**Authors:** Ralph N Martins, Purna Poudel, Shaun Frost, Shaun Eslick, Hamid R Sohrabi, Kevin Taddei, Eugene Hone

**Affiliations:** ^1^ Edith Cowan University, Perth, Western Australia, Australia; ^2^ Australian Alzheimer's Research Foundation, Perth, Western Australia, Australia; ^3^ CSIRO, Perth, Western Australia, Australia; ^4^ Macquarie University, Macquarie Park, NSW, Australia; ^5^ Murdoch University, Murdoch, Western Australia, Australia; ^6^ Edith Cowan University, Joondalup, Australia; ^7^ Alzheimer's Research Australia, Perth, Western Australia, Australia; ^8^ Lions Alzheimer's Foundation, Perth, Western Australia, Australia

## Abstract

**Background:**

Dementia is currently the second leading cause of death in Australia and the seventh leading cause of death worldwide. Diagnosis of Alzheimer's disease (AD), the major cause of dementia, is difficult and time consuming. Current clinical imaging technologies are costly to use for widespread early screening of AD and have limited availability. In contrast, the retina is unique, where blood vessels and neural tissue can be viewed and imaged non‐invasively and relatively inexpensively. As part of the central nervous system, the retina exhibits similarities to the brain and can display indicators of various neurological disorders, including AD. We aimed to image the retina and analyse its spectral features to develop a robust machine learning (ML) classification model that distinguishes between brain amyloid‐beta (Aβ) positive and negative individuals.

**Method:**

Sixty‐eight consenting volunteers with varying levels of brain Aβ protein underwent non‐invasive imaging using a hyperspectral retinal camera and illumination of wavelengths from 450 to 905 nm. Multiple retina features from the central and superior views were selected and analysed to identify their variability among individuals with different brain amyloid loads. Eight ML models were evaluated for their performance in predicting brain Aβ levels using the retina images and systemic factors like age, gender and apolipoprotein E (APOE) genotype.

**Result:**

The retinal reflectance spectra in the 405–585 nm wavelengths exhibited a significant difference in individuals with increasing brain amyloid. The retinal features in the superior view showed higher inter‐subject variability. Our comparison of eight different ML algorithms revealed that the Multi‐Layer Perceptron (MLP) model exhibited superior classification performance, achieving an accuracy of 0.86, precision of 0.88, recall of 0.82, F1‐score of 0.85, and area under curve (AUC) of 0.90.

**Conclusion:**

This study indicates that there are spectral variations of retinal features associated with brain amyloid loads. It also demonstrates the feasibility of the retinal hyperspectral imaging (rHSI) technique as a potential non‐invasive, inexpensive screening method to identify individuals in the preclinical phase of AD.

